# *Leishmania amazonensis* from distinct clinical forms/hosts has polymorphisms in Lipophosphoglycans, displays variations in immunomodulatory properties and, susceptibility to antileishmanial drugs

**DOI:** 10.1002/cbin.11880

**Published:** 2022-08-23

**Authors:** Felipe D. Rêgo, Camila d. A. Cardoso, Paulo Otávio L. Moreira, Paula M. Nogueira, Márcio S. Araújo, Valéria Matos Borges, Márcia D. Laurenti, Daniella C. Bartholomeu, Alexandre B. Reis, Rubens L. d. Monte‐Neto, Rodrigo P. Soares

**Affiliations:** ^1^ Biotechnology Applied to Pathogens (BAP), Instituto René Rachou Fundação Oswaldo Cruz (FIOCRUZ) Belo Horizonte MG Brazil; ^2^ Laboratory of Inflammation and Bioarkers, Instituto Gonçalo Muniz Fundação Oswaldo Cruz (FIOCRUZ) Salvador BA Brazil; ^3^ Departamento de Patologia, Faculdade de Medicina Universidade de São Paulo (USP) São Paulo SP Brazil; ^4^ Departamento de Parasitologia Universidade Federal de Minas Gerais (UFMG) Belo Horizonte MG Brazil; ^5^ Núcleo de Pesquisas em Ciências Biológicas Universidade Federal de Ouro Preto (UFOP) Ouro Preto MG Brazil

**Keywords:** antileishmanial agents, glycoconjugates, innate immunity, *Leishmania amazonensis*, lipophosphoglycan, macrophage

## Abstract

Lipophosphoglycan (LPG), the major *Leishmania* glycoconjugate, induces pro‐inflammatory/immunosuppressive innate immune responses. Here, we evaluated functional/biochemical LPG properties from six *Leishmania amazonensis* strains from different hosts/clinical forms. LPGs from three strains (GV02, BA276, and LV79) had higher pro‐inflammatory profiles for most of the mediators, including tumor necrosis factor alpha and interleukin 6. For this reason, glycoconjugates from all strains were biochemically characterized and had polymorphisms in their repeat units. They consisted of three types: type I, repeat units devoid of side chains; type II, containing galactosylated side chains; and type III, containing glucosylated side chains. No relationship was observed between LPG type and the pro‐inflammatory properties. Finally, to evaluate the susceptibility against antileishmanial agents, two strains with high (GV02, BA276) and one with low (BA336) pro‐inflammatory activity were selected for chemotherapeutic tests in THP‐1 cells. All analyzed strains were susceptible to amphotericin B (AmB) but displayed various responses against miltefosine (MIL) and glucantime (GLU). The GV02 strain (canine visceral leishmaniasis) had the highest IC_50_ for MIL (3.34 μM), whereas diffuse leishmaniasis strains (BA276 and BA336) had a higher IC_50_ for GLU (6.87–12.19 mM). The highest IC_50_ against MIL shown by the GV02 strain has an impact on clinical management. Miltefosine is the only drug approved for dog treatment in Brazil. Further studies into drug susceptibility of *L. amazonensis* strains are warranted, especially in areas where dog infection by this species overlaps with those caused by *Leishmania infantum*.

## 1 INTRODUCTION

*Leishmania* infections result in a spectrum of clinical manifestations determined by complex host‐parasite interactions. *Leishmania amazonensis* has been identified from patients with diverse clinical forms of leishmaniasis including localized cutaneous leishmaniasis (LCL), anergic diffuse (ADCL), muco‐cutaneous (MCL), and canine visceral leishmaniasis (CVL) in South American countries, mainly in Brazil (Lainson et al., 1994; F. T. Silveira et al., 2004). Among the cutaneous forms, ADCL is the most severe as therapeutic failures are common. A distinguished feature of this form is an impairment in cellular responses, causing T cells anergy and lack of delayed‐type hypersensitivity (Convit et al., 1972; Desjeux, 2004; F. T. Silveira et al., 2005). The anergic nature of *L. amazonensis* remains obscure, although several mechanisms have been suggested (Real et al., 2013).

The reduced incidence of the number of *L. amazonensis* LCL cases in the north and northeast of Brazil, where this parasite is distributed, may be a result of its zoonotic and occupational patterns (Camara Coelho et al., 2011; J. P. de Oliveira et al., 2007). This species is transmitted by *Bichromomyia flaviscutellata*, a sand fly species found in the ground of forested areas, having wild rodents as hosts (Lainson & Shaw, 1968). Bats have also been suggested as opportunistically involved in wild cycles outside the Amazon region (E. F. de Oliveira et al., 2015; Savani et al., 2010). *B. flaviscutellata* is widely distributed in the Amazon region and other Brazilian states (Carvalho et al., 2015). In Minas Gerais, southeastern Brazil, *L. amazonensis* was found in wild‐caught sand flies (M. S. Cardoso et al., 2019; Rêgo et al., 2015) and in dogs, causing CVL (Dias et al., 2011; Valdivia et al., 2017). This finding is of particular concern since in 2017, the treatment of dogs with miltefosine (MIL) was approved in Brazil. Most CVL cases are caused by *Leishmania infantum* and the natural resistance of *L. amazonensis* to antileishmanial drugs (Bittencourt et al., 1989; Convit et al., 1989) may lead to therapeutic failure. However, the drug susceptibility profile from a viscerotropic *L. amazonensis* causing CVL is unknown.

The exuberant growth of *L. amazonensis* in culture facilitated its use as a model species for immunology and chemotherapy (Rocha et al., 2013; Rodrigues et al., 2010). The classical TH1/TH2 phenotype, observed for *Leishmania major* in C57BL/6 and BALB/c mice, is not followed by *L. amazonensis*. It causes severe cutaneous lesions in both mice with a mixed cytokine profile (Pereira & Alves, 2008). Several reports attempted to elucidate *Leishmania* virulence factors during infection, especially those involving the parasite glycoconjugates. Lipophosphoglycan (LPG), the major cell surface glycoconjugate of *Leishmania*, has been implicated in a wide range of functions (de Assis et al., 2012). Regarding dermotropic species, functional studies of *L. amazonensis* LPGs have shown their role in macrophages and neutrophils. Those include induction of neutrophil extracellular traps (Guimarães‐Costa et al., 2009), double‐stranded RNA‐dependent protein kinase (PKR) (de Carvalho Vivarini et al., 2011), LTB_4_ (Tavares et al., 2014), NO/cytokines via TLR4 (Nogueira et al., 2016), caspase‐11 via NLRP3 (de Carvalho et al., 2019), and IL‐32 via TLR2/NOD2 (Silveira et al., 2022). However, an unknown aspect of *L. amazonensis* glycobiology is to what extent LPG polymorphisms from different strains may functionally affect macrophage responses.

LPG structures have been described as several dermotropic and viscerotropic *Leishmania* species worldwide. Inter‐ and intraspecies polymorphisms are usually found in the sugars branching off the conserved Gal(β1,4)Man(α1)‐PO_4_ motif (de Assis et al., 2012). However, studies focusing on LPG intraspecies polymorphisms have used a limited number of strains (Mahoney et al., 1999; Nogueira et al., 2017; Paranaíba et al., 2015; Soares et al., 2004). In the past few years, some studies have increased the panel of strains from different clinical manifestations/hosts. LPGs from viscerotropic/dermotropic *L. infantum* possess three types of LPG: (I) without side chains, (II) with one β‐glucose linked to the repeat units, and (III) with two to three β‐glucoses as side chains. Those polymorphisms did not affect sand fly development but affected NO/cytokine production by murine macrophages (Cardoso et al., 2020; Coelho‐Finamore et al., 2011; Soares et al., 2002). Like *L. infantum*, *Leishmania braziliensis* LPGs from different clinical forms also displayed unbranched and branched sugars in their repeat units. These polymorphisms did not correlate with NO and cytokine production by murine macrophages (Vieira et al., 2019). Finally, preliminary reports on *L. amazonensis* LPG showed glycosylated and galactosylated side chains in the strains PH8 and Josefa, isolated from sand flies and humans, respectively. These LPGs were potent TLR4 agonists and induced NO and cytokine production by murine macrophages. However, they did not affect sand fly interaction with *Migonemyia migonei* and *Lutzomyia longipalpis* (Nogueira et al., 2016, 2017).

As part of a wider project on the glycobiology of *Leishmania* parasites, we evaluated the role of *L. amazonensis* LPGs from distinct clinical forms/hosts during interaction with murine macrophages. Since we have a valuable panel of strains, we also evaluated their susceptibility profile against antileishmanial drugs.

## 2 MATERIALS AND METHODS

### 2.1 Parasite culture and molecular typing

Six strains of *L. amazonensis* were evaluated (Table 1). Glycobiology experiments used *L. amazonensis* reference strain (IFLA/BR/1967/PH8) as control (Nogueira et al., 2017). Promastigotes were cultured in M199 medium supplemented with 10% fetal bovine serum (FBS) (Invitrogen/Thermo Fisher Scientific, penicillin, 200 u/ml), and streptomycin (200 μg/mL) (all Merck KGaA), at 25°C. To confirm parasite identity, molecular typing (*hsp*70 gene) was performed (Garcia et al., 2004). Confirmed *L. amazonensis* sequences were deposited in the GenBank database (accession numbers OM780131‐OM780136).

**Table 1:** *Leishmania amazonensis* strains isolated from distinct clinical forms and hosts

Strain	Nomenclature used in the text	Clinical form	Host	Origin
IFLA/BR/1967/PH8	PH8	ND	*B. flaviscutellata*	Pará/BR
MHOM/BR/1987/BA115	BA115	LCL	*Homo sapiens*	Bahia/BR
MHOM/BR/1987/BA125	BA125	LCL	*H. sapiens*	Bahia/BR
MHOM/BR/1987/BA276	BA276	ADCL	*H. sapiens*	Bahia/BR
MHOM/BR/1989/BA336	BA336	ADCL	*H. sapiens*	Bahia/BR
MCAN/BR/2012/GV02	GV02	CVL	*Canis familiaris*	Minas Gerais/BR
MPRO/BR/72/M1845	LV78	ND	*Proechimys* sp.	Pará/BR

Abbreviations: ADCL, anergic diffuse cutaneous leishmaniasis; *B. Flaviscutellata*, *Bichromomyia flaviscutellata*; CVL, canine visceral leishmaniasis; LCL, localized cutaneous leishmaniasis; ND, not determined.

### 2.2 Extraction, purification, and quantitation of LPG

**Figure 1:**
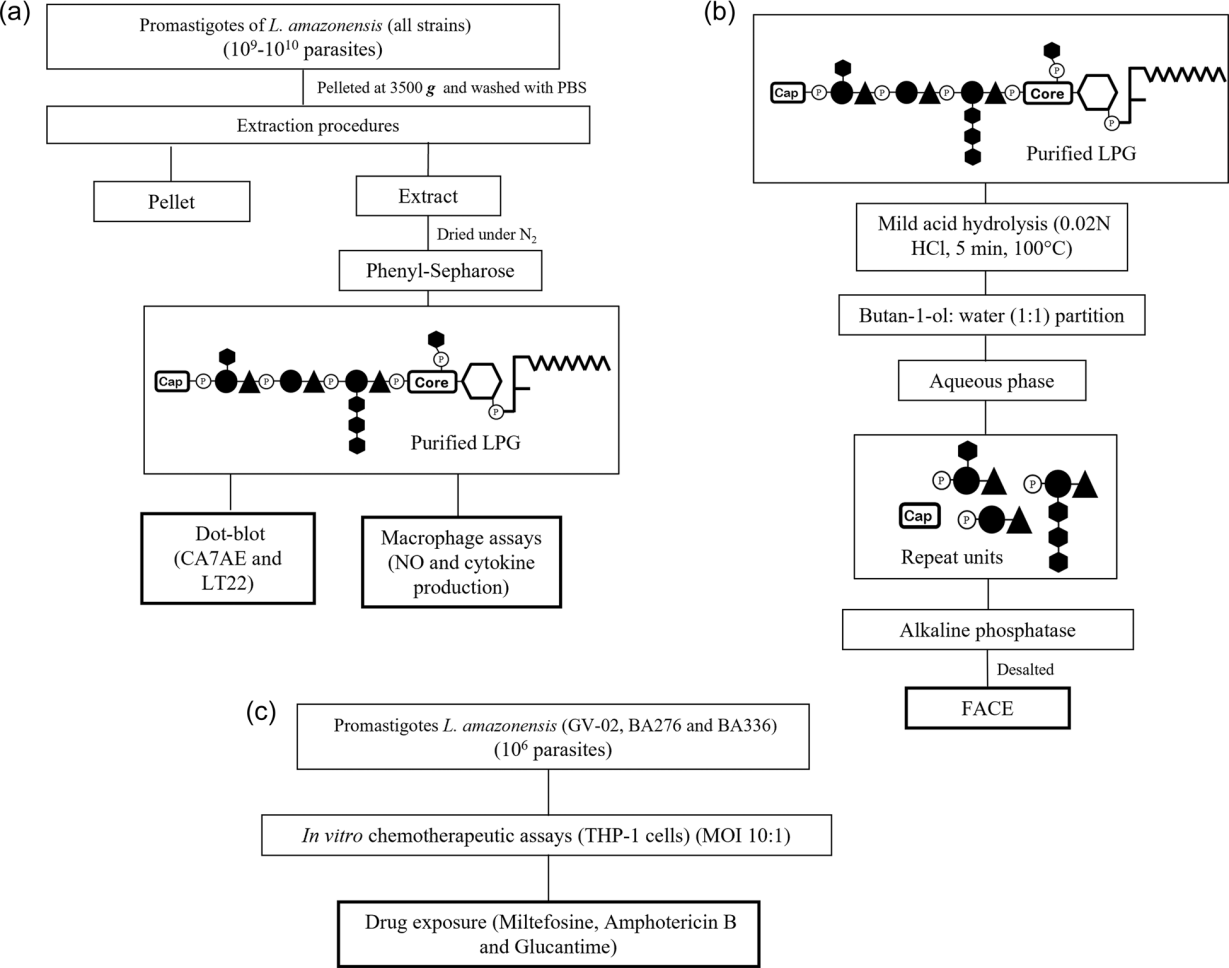
Strategies employed for *Leishmania amazonensis* strains characterization. (a) Extraction, purification, dot‐blots, and interaction with murine macrophages. Parasite cell pellets were subject to extraction with organic solvents as described elsewhere. For purification, the solvent E extract was dried under N_2_ evaporation and applied into a phenyl‐Sepharose column. Purified lipophosphoglycan (LPGs) were used for biological and immunological assays. (b) Biochemical characterization of LPG repeats units by fluorophore‐assisted carbohydrate electrophoresis (FACE). LPG was depolymerized, subjected to butanol:water partition and treated with alkaline phosphatase. After desalting, neutral repeat units were subjected to FACE. (c) Chemotherapeutic assays. THP‐1 cells were exposed to parasites (MOI 10:1) before drug exposure and IC_50_ determination.

LPGs were extracted with organic solvents and purified using phenyl‐Sepharose from late log‐phase cells (Nogueira et al., 2017). Organic eluates were dried through N_2_ evaporation and purified LPGs were resuspended in endotoxin–free water (Sanobiol) and quantitated using the phenol‐sulfuric method (Dubois et al., 1956). The LPG concentrations were adjusted to 10 μg/mL in RPMI before functional experiments (Nogueira et al., 2016) (Figure 1a).

### 2.3 Functional assays

To evaluate the functional properties of LPGs, they were added to murine macrophages. Intraperitoneal macrophages previously stimulated with 2 ml of 3% sterile thioglycolate were extracted from BALB/c mice through sequential washing with cold RPMI without supplement. Cell viability was checked by trypan blue. Cells (3 × 10^5^/well) were seeded into 96‐well culture plates for adhesion (1 h, 37°C, 5% CO_2_) with RPMI supplemented with 2 mM glutamine, 50 U/ml of penicillin, 50 μg/ml streptomycin, and 10% FBS. Cells were primed with interferon‐gamma (IFN‐γ) (3 U/ml) for 18 h before incubation with LPGs (10 μg/ml) from each strain and lipopolysaccharide (LPS) (100 ng/ml, positive control) (48 h, 37°C, 5% CO_2_) (Figure 1a).

### 2.4 Cytokine, chemokine, and nitrite measurements

To evaluate the production of different mediators in response to LPG and LPS, macrophage culture supernatants were collected after 48 h of incubation. Tumor necrosis factor alpha (TNF‐α), interleukin 6 (IL‐6), IL‐10, IL‐12p70, and MCP‐1 concentrations were determined using BD cytometric bead array (CBA) Mouse Inflammation Kit (BD Biosciences) according to the manufacturer's specifications. Flow cytometry measurements were performed on FACSVerse flow cytometry (BD Biosciences). The Cell‐Quest software package provided by the manufacturer was used for data acquisition and the FlowJo v. 7.6.4 (Tree Star Inc.) was used for data analysis. A total of 2500 events were acquired for each analysis. The results are representative of three experiments in duplicate. Nitrite (NO) concentrations were determined by the Griess reaction (Drapier et al., 1988).

### 2.5 Fluorophore‐assisted carbohydrate electrophoresis (FACE)

As LPGs have differently activated murine macrophages, our next step was to evaluate the existence of sugar polymorphisms in their repeat units. LPGs were depolymerized after mild acid hydrolysis and phosphorylated repeat units were recovered after butanol:water partition (Soares et al., 2002). Then, they were treated with alkaline phosphatase (1 U) in 15 mM Tris buffer (pH 9.0, 16 h, 37°C). Samples were desalted by passage through a two‐layered column of AG50W‐X12 (H+) over AG1‐X8 (acetate) and fluorescently labeled with 0.05 N ANTS (8‐aminonaphthalene‐1,3,6‐trisulfate) and 1 M cyanoborohydride for 16 h, 37°C (Soares et al., 2004). After this step, samples were subjected to FACE analysis, including the labeled oligo‐glucose ladders (G_1_–G_7_) used as standards. For monosaccharide analysis, the repeat units were subjected to strong acid hydrolysis (2 N trifluoroacetic acid, 3 h, 100°C). Samples were desalted as described above and fluorescently labeled with 0.1 M AMAC (2‐aminoacridone) in 5% acetic acid and 1 M cyanoborohydride. Then, monosaccharides were also subjected to FACE. Poly‐ and monosaccharide gels were visualized under UV exposure (Nogueira et al., 2017) (Figure 1b).

### 2.6 Immunoblotting

To confirm the quality of the side chains observed in the FACE analysis, purified LPGs were subjected to dot‐blot analysis. LPG (2 µg) was blotted onto a nitrocellulose membrane (Amersham Protran, 0.45 μm—GE Healthcare), blocked (1 h) in 5% powdered and skimmed milk (Molico, Nestlé), and probed for 18 h with mAb CA7AE (1:1000) that recognizes the Gal(β1,4)Man(α1)‐PO_4_ epitope (Tolson et al., 1994) and mAb LT22 (1:1000), that recognizes β‐glucose side chains (Lira et al., 1998). Then, the membrane was washed three times in PBS (3 × 5 min) and incubated with anti‐mouse IgG conjugated with peroxidase (1:10,000) for 1 h. After a final wash with PBS (3 × 5 min), the reaction was visualized with luminol (Guimarães et al., 2018).

### 2.7 Macrophage experimental infection and drug susceptibility assay

To evaluate the susceptibility of *L. amazonensis* strains to current antileishmanial drugs, chemotherapeutic assays were performed as previously reported (Rugani et al., 2018). Briefly, human monocyte‐derived macrophages from THP‐1 cell line (ATCC#TIB‐202) were infected with 2 × 10^5^ stationary‐phase promastigotes (MOI 10:1) for 3 h. Noninternalized parasites were removed, and cells were incubated for 72 h in the presence/absence (untreated control) of amphotericin B (AmB), MIL, and glucantime (GLU). Assays were performed twice in three replicates. The infection index was obtained by dividing the total number of infected cells at each drug concentration by the number of infected cells from the untreated control. The mean number of amastigotes was represented by the total of amastigotes per 100 macrophages and the number of amastigotes per macrophage was obtained by dividing the number of intracellular amastigotes by the total of infected host cells (Figure 1c).

### 2.8 Statistical analysis

For nitrite, cytokine, and chemokine measurements, the Shapiro–Wilk normality test was conducted to test the null hypothesis that data were sampled from a Gaussian distribution. Ordinary one‐way analysis of variance was performed to compare the nitrite, cytokines, and chemokine productions among *L. amazonensis* strains. The data were analyzed using GraphPad Prism 7.0 software (Graph Prism Inc.). The half‐maximal inhibitory concentration (IC_50_) of the antileishmanial drugs was calculated based on the infectivity index profile where log transformed drug concentration values versus normalized responses with variable slopes, were applied in sigmoidal dose–response curves, performed using GraphPad Prism version 7.0 software (Graph Prism Inc.). The *p* values below .05 were considered statistically significant.

## 3 RESULTS

### 3.1 Functional analysis

**Figure 2:**
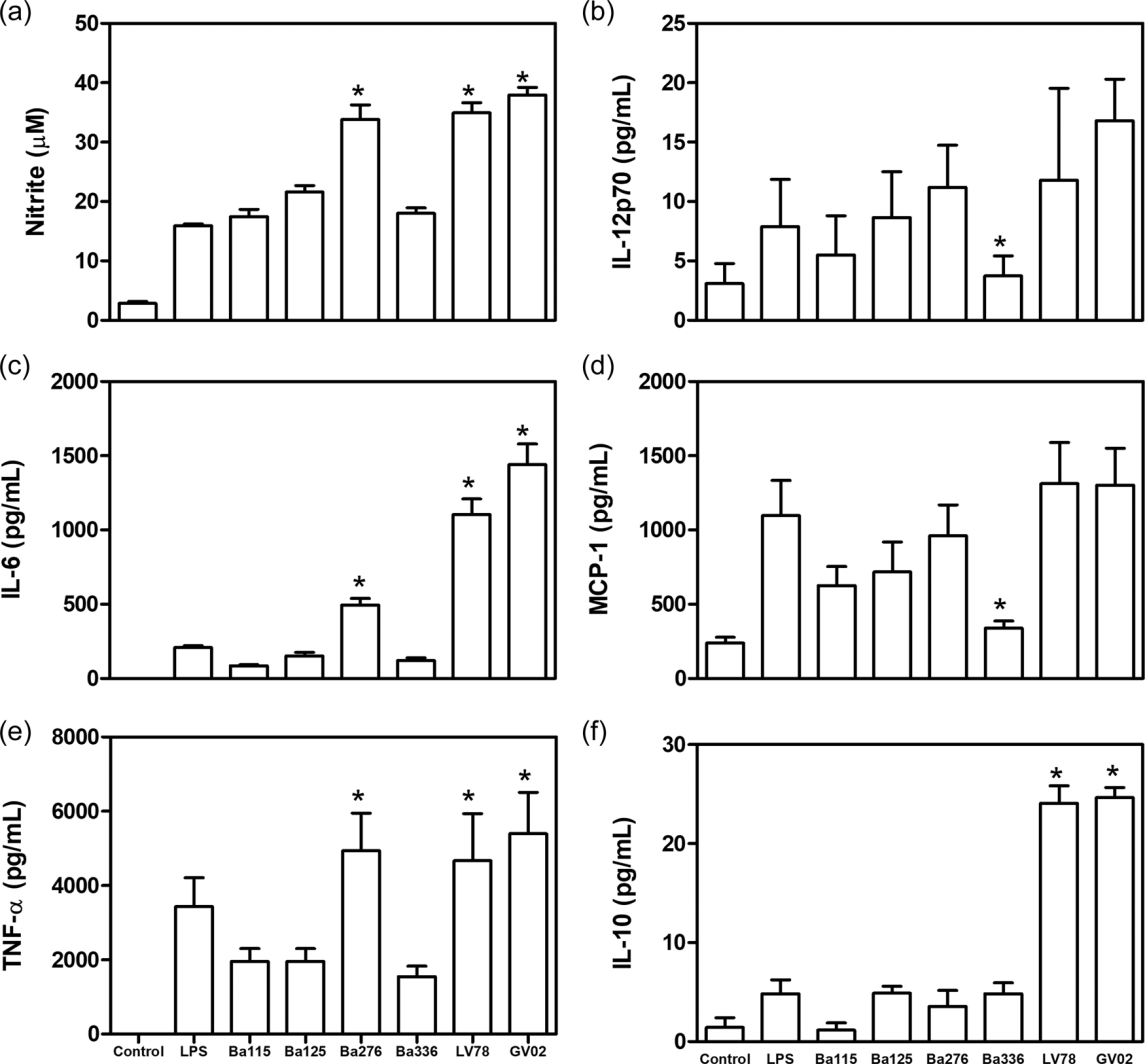
Nitrite (a) and cytokines/chemokine (b–f) production by interferon‐gamma primed murine macrophages stimulated with lipophosphoglycan (LPGs) from distinct *Leishmania amazonensis* strains. Nitrite concentration was measured by Griess reaction and cytokine concentrations were determined by flow cytometry. Negative control and positive control were medium and lipopolysaccharide (100 ng/ml), respectively. Asterisks indicate statistical differences (*p* < .05).

LPGs were able to differentially stimulate the production of different mediators by peritoneal murine macrophages (Figure 1). LPGs from BA276, LV78, and GV02 strains induced higher levels of NO, IL‐6, and TNF‐α than the others (*p* < .05) (Figure 2a–c). Those levels were even higher than that for LPS (positive control). The heterogeneous production of IL‐12p70 and MCP‐1 was observed among strains being statistically higher than the BA336 strain (Figure 2d,e). Finally, IL‐10 production was higher for GV02 and LV78 LPGs only (Figure 2f).

### 3.2 LPG polymorphisms in *L. amazonensis* strains

**Figure 3:**
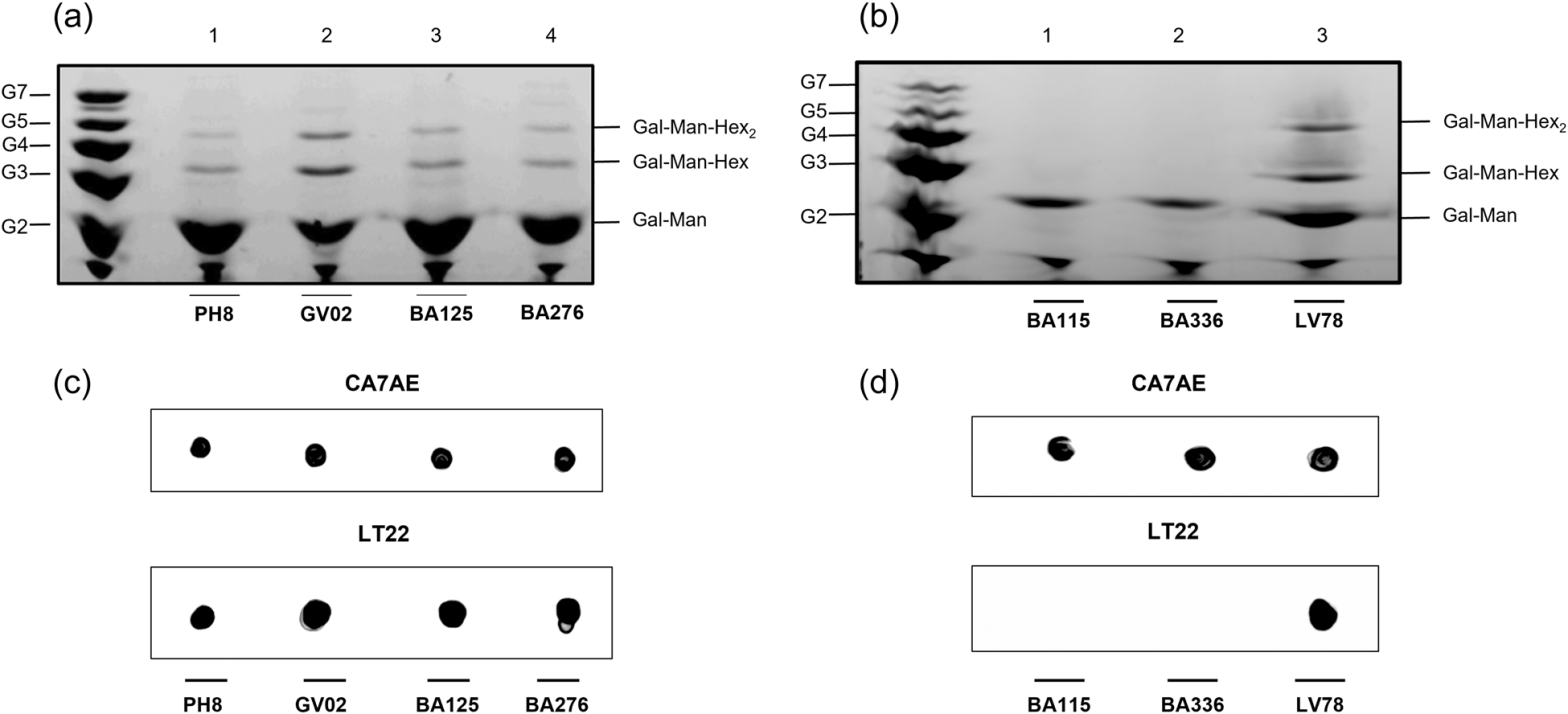
Biochemical characterization of lipophosphoglycan (LPGs) from distinct *Leishmania amazonensis* strains. Fluorophore‐assisted carbohydrate electrophoresis of dephosphorylated repeat units. (a) G_2_–G_7_, oligoglucose ladder; lane 1, PH8 strain; lane 2, GV02 strain; lane 3, BA125 strain and lane 4, BA276 strain. (b) G_2_–G_7_, oligoglucose ladder; lane 1, BA115 strain; lane 2, BA336 strain and lane 4, LV78 strain. (c, d) Dot blot analysis of intact LPG using mAbs (1:1000) CA7AE (upper), and LT22 (lower).

To investigate if functional variations could be due explained by the LPG polymorphisms, these molecules were biochemically characterized. They displayed qualitative differences in their repeat units by FACE analysis that were further and confirmed by the dot‐blot analysis (Figure 3). Carbohydrate profiles showed the presence of the disaccharide Gal(β1,4)Man(α1) (G_2_), and one to two side chains (G_3_–G_4_) in the repeat units of GV02, BA125, BA276, and LV78 strains (Figure 3a, lanes 2–4 and; Figure 3b, lane 3). The repeat units of BA115 and BA336 strains were devoid of side chains and co‐migrated with the disaccharide G_2_, confirming the structure of Gal(β1,4)Man(α1) common to all LPGs (Figure 3b, lanes 1 and 2). As expected, the repeat units of the PH8 strain (control) showed 1‐2 β‐glucoses as side chains (G_3_–G_4_) branching‐off the disaccharide Gal(β1,4)Man(α1) (G_2_) as previously reported (Nogueira et al., 2017) (Figure 3a, lane 1).

**Figure 4:**
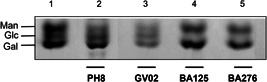
Fluorophore‐assisted carbohydrate electrophoresis (FACE) of monosaccharides from distinct *Leishmania amazonensis* strains. hydrolysis. Lane 1, monosaccharide standards; lane 2, control strain (PH8); lane 3, (GV02); lane 4, BA125, and, lane 5, BA276). Man, mannose; Gal, galactose; Glc, glucose.

To confirm the quality of the hexoses branching‐off the repeat units of the LPGs from GV02, BA125, BA276, and, LV79, two mAbs were used: mAb CA7AE, which recognizes the unsubstituted disaccharide Gal(β1,4)Man(α1) (Figure 2c,d, upper) and LT22, specific for glucose/galactose side chains (Figure 2c,d, lower). Consistent with the FACE analysis, all strains were recognized by CA7AE, confirming the structure of Gal(β1,4)Man(α1) (G_2_) common to all LPGs. However, LT22 only recognized branched structures in the LPGs from strains PH8 (control), GV02, BA125, Ba276, and LV78 (Figure 2c,d, lower). Since LPGs from *L. amazonensis* have either galactose or glucose as side chains, monosaccharide analysis was performed in four strains to confirm the quality of sugars branching‐off the repeat units (Figure 4). Confirming previous data, the repeat units of PH8 (control) showed expected galactose, mannose, and a glucose band (Figure 4, lane 2) (Nogueira et al., 2017). Like PH8, the monosaccharide profile of the GV02 strain also had glucose as side chains (Figure 4, lane 3). Finally, BA125 and BA276 LPGs had only galactose and mannose in their monosaccharide content (Figure 4, lanes 4 and 5). However, the higher intensity of the galactose band implies that this sugar is present in their side chains. Altogether, those data confirmed intraspecies polymorphisms in the LPGs of *L. amazonensis* strains bearing galactose and glucose as side chains and, unbranched repeat units. The proposed LPG repeat units for different *L. amazonensis* strains (types I–III) from different clinical forms/hosts are summarized in Table 2.

**Table 2:** Proposed LPG structures (types I–III) of *Leishmania amazonensis* strains from different clinical forms and hosts

Strain	Clinical form	Side chain	Type
MHOM/BR/1987/BA115	LCL	None	I
MHOM/BR/1989/BA336	ADCL	None	I
MHOM/BR/1975/Josefa^a^	LCL	1–3 galactoses	II
MHOM/BR/1987/BA125	LCL	1–2 galactoses	II
MHOM/BR/1987/BA276	ADCL	1–2 galactoses	II
IFLA/BR/1967/PH8^a^	ND	1–2 glucoses	III
MCAN/BR/2012/GV02	CVL	1–2 glucoses	III
MPRO/BR/72/M1845	ND	1–2 hexoses^b^	II or III

Abbreviations: ADCL, anergic diffuse cutaneous leishmaniasis; FACE, fluorophore‐assisted carbohydrate electrophoresis; CVL, canine visceral leishmaniasis; LCL, localized cutaneous leishmaniasis; LPG, lipophosphoglycan; ND, not determined.

^a^ Structures extracted from Nogueira et al. (2017).

^b^ Based on FACE analysis and reactivity to LT22.

### 3.3 Susceptibility to antileishmanial drugs

**Figure 5:**
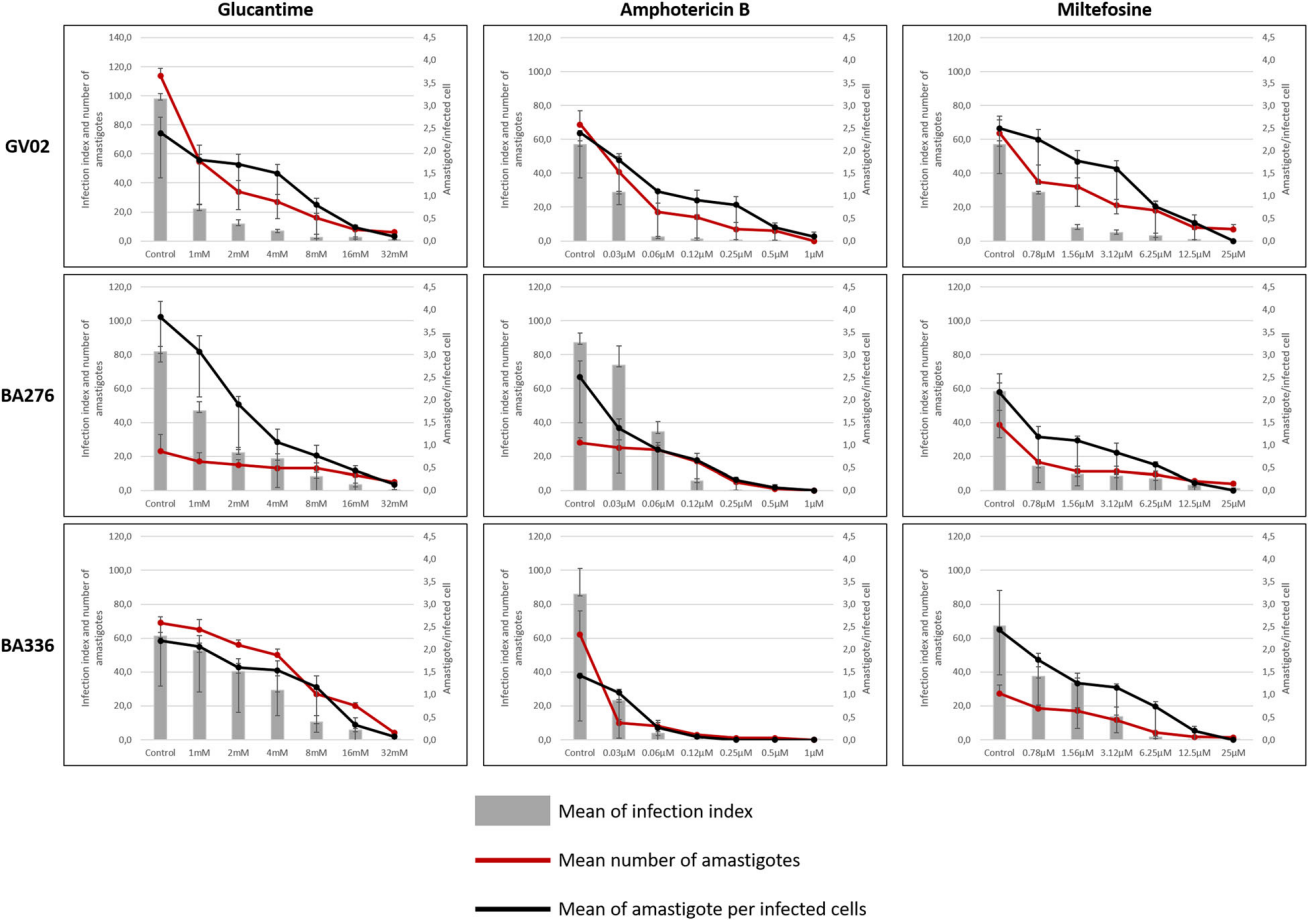
Susceptibility profile of GV02 BA276 and Ba336 strains of *Leishmania amazonensis* to current antileishmanial agents. Bars indicate the percentage of infection of monocyte‐derived human THP‐1 macrophages (*y* axis) in serial‐diluted drug concentrations (*x* axis). Red lines indicate the mean number of amastigotes per 100 macrophages and the black lines represent the mean number of amastigotes per macrophage. The data refer to an average of three independent experiments, with error bars representing the standard error of the mean.

**Figure 6:**
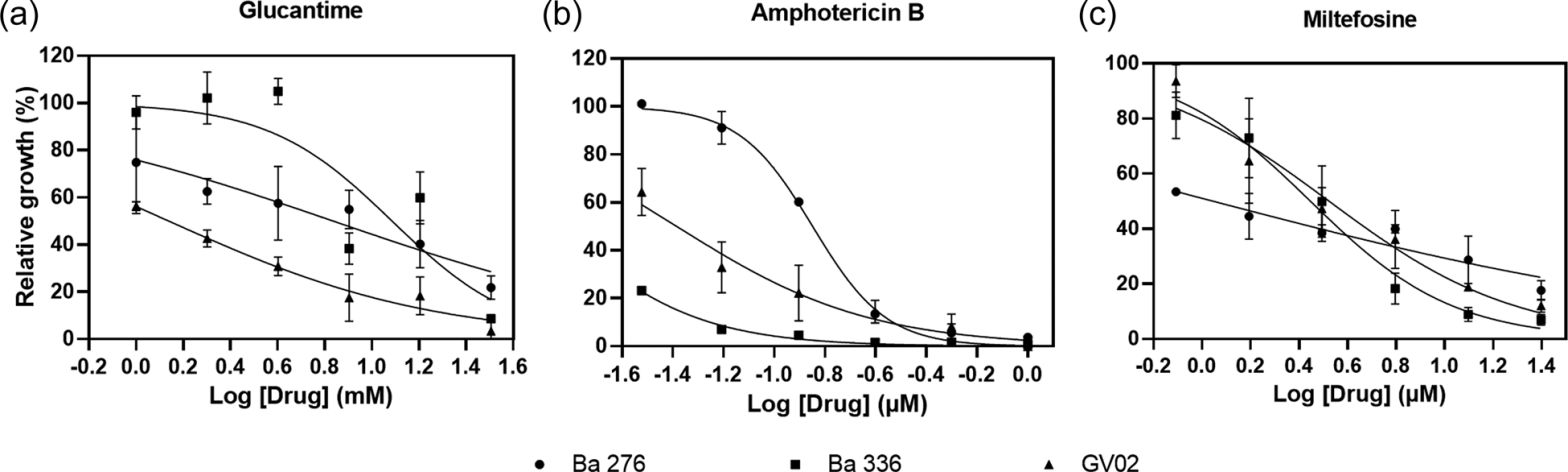
Susceptibility profile of GV02 BA276 and Ba336 strains of *Leishmania amazonensis* strains to antileishmanial agents. Drug curve plots are shown as mean and standard deviation in triplicate.

As *L. amazonensis* strains elicited distinct immunomodulatory responses on macrophage experimental infection, we investigated the drug susceptibility status of three strains: one isolated from a dog (GV02) and two (BA276 and BA336) isolated from ADCL patients that are often resistant to available antileishmanial protocols (Zauli‐Nascimento et al., 2010). All strains were sensitive to GLU, AmB, and MIL in a dose‐dependent manner (Figure 5). However, intraspecies variations in IC_50_ values showed different susceptibility profiles against antileishmanial drugs (Table 3, Figure 6). As expected, all strains were equally susceptible to AmB showing the lowest IC_50_ values. However, ACDL strains (BA276 and BA336) had higher IC_50_ values than CVL (GV02). Conversely, GV02 strain had a higher (~3‐fold) IC_50_ value for MIL (Table 3).

**Table 3:** Half inhibitory concentrations (IC_50_) of antileishmanial drugs against intracellular amastigotes of *L. amazonensis* from different strains

Samples	Clinical form	IC_50_ (CI 95%)		
		Glucantime (mM)	Amphotericin B (µM)	Miltefosine (µM)
BA276	ADCL	6.87 (4.12–11.36)	0.14 (0.13–0.15)	1.11 (0.54–1.72)
BA336	ADCL	12.19 (8.33–18.32)	0.014 (0.011–0.016)	2.80 (2.32–3.35)
GV02	CVL	1.38 (1.01–1.75)	0.041 (0.029–0.052)	3.34 (2.66–4.19)

Abbreviations: ADCL, anergic diffuse cutaneous leishmaniasis; CVL, canine visceral leishmaniasis.

## 4 DISCUSSION

Several factors affect infectivity and/or pathogenicity among *Leishmania* strains. In *L. amazonensis*, factors favoring its ability to cause a wide spectrum of clinical manifestations are unknown. Parasite surface glycoconjugates are important virulence factors (De Assis et al., 2012; Sacks & Kamhawi, 2001). However, we do not know in which extent they contribute for intraspecies variations and clinical outcomes. Preliminary observations reported LPG polymorphisms in two *L. amazonensis* strains (PH8 and Josefa) (Nogueira et al., 2016, 2017). Although LPG polymorphisms were evident, they did not trigger differential immune responses in macrophages. Here, to better address this subject, the number of *L. amazonensis* strains was expanded. The functional properties of their LPGs and susceptibility to current antileishmanial drugs were evaluated.

Functionally, *L. amazonensis* LPGs were able to trigger distinct pro‐inflammatory innate immune responses. The *Leishmania* LPGs from dermotropic species/strains can usually induce higher NO/cytokines production than viscerotropic ones (Cardoso et al., 2020; Ibraim et al., 2013; Nogueira et al., 2016; Paranaíba et al., 2015; Vieira et al., 2019). When an increased number of strains is used, variations in their pro‐inflammatory/immunosuppressive LPG properties were documented (Cardoso et al., 2020; Coelho‐Finamore et al., 2011; Vieira et al., 2019). Consistent with these observations, *L. amazonensis* also followed this pattern. With exception of IL‐10 (BA276), higher induction of NO, IL‐6, TNF‐α, and IL‐10 were observed for BA276 (ADCL), LV78 (rodent), and GV02 (CVL) strains. In some cases, this induction was even higher than that caused by LPS. Overall, IL‐10 is a pleiotropic immunomodulatory cytokine suppressing Th1‐dependent cell‐mediated immunity and increasing TH2 immune responses (De Waal Malefyt et al., 1991; Fernandez‐Botran et al., 1988). High IL‐10 levels in the initial phase of VL lead to susceptibility of infection by decreasing the frequency of multifunctional CD4 T cells (Mesquita et al., 2018). Here, highest IL‐10 levels for rodent (LV78) and canine (GV02) were detected. VL pathogenesis appears at least in part due to a shift in the balance of effector/regulatory mechanisms. In this specific case, the higher IL‐10 level triggered by *L. amazonensis* LPG from a canine strain may contribute to an inefficient TH1 response.

On the other hand, BA336 (causing ADCL) LPG, was a poor inducer of IL‐12 and MCP‐1. IL‐12 is a key cytokine promoting cellular activation during *Leishmania* infection, leading to a TH1‐type response (Lohoff et al., 1999). Although this finding was interesting, it could not be correlated to anergy since the LPG from another ADCL strain (BA276) induced higher levels of this cytokine. Based on the pro‐inflammatory properties of the LPGs, a clear correlation between clinical form/host was not noticed. Our next step was to check for LPG polymorphisms affecting macrophage activation.

Consistent with our previous reports, polymorphisms in repeat units were detected and considered to be of three types: Some were devoid of side chains (type I), others had 1–2 β‐galactoses (type II) or 1–2 β‐glucoses (type III) as side chains (summarized in Table 2). Type II and III structures were already reported for *L. amazonensis* (Nogueira et al., 2017). Here, we reported for the first‐time unbranched repeat units (type I) in two dermotropic strains BA115 (LCL) and BA336 (ADCL). Type I LPG is found in most *L. infantum* strains (Coelho‐Finamore et al., 2011), *L. braziliensis* (Soares et al., 2005; Vieira et al., 2019), and *Leishmania shawi* (Passero et al., 2015). Type II repeat units were detected in dermotropic strains BA125 (LCL) and BA276 (ADCL). Poly‐galactosylated LPGs were already reported in *L. major* (Dobson et al., 2003; McConville et al., 1992), and *Leishmania tropica* (Soares et al., 2004). Finally, type III (glucosylated) LPG were found in GV02 and PH8 strains. This type of LPG has already been reported for *L. infantum* (Coelho‐Finamore et al., 2011) and *Leishmania donovani* (Mahoney et al., 1999). Confirming previous functional studies, polymorphisms in *L. amazonensis* LPGs did not correlate with clinical forms/hosts. For example, carbohydrate motifs of ADCL and LCL strains were identical. This suggests that functional LPG abilities are strain specific. In summary, our panel of *L. amazonensis* strains displayed functional/biochemical variations. This species is often resistant to antileishmanial drugs, and we have one unique strain isolated from a canine infection. Our next step was to evaluate intraspecies susceptibility to several antileishmanial agents.

Although *L. infantum* is the main CVL species in Brazil, detection of *L. amazonensis* causing similar symptoms in dogs is worrisome. All strains were sensitive to AmB, but differences in IC_50_ values were detected for GLU and MIL. For example, the ADCL strain exhibited higher (~7‐ to 12‐fold) IC_50_ values than the GV02, reinforcing field observations on antimonial therapeutic failure in human patients. Conversely, for MIL, a higher (~1.3‐fold) IC_50_ value was observed for the GV02 strain. Although this difference was within a range closer to the ADCL strains, it shows that the GV02 strain is slightly more resistant to MIL. Miltefosine is the only approved drug for CVL treatment in Brazil and its efficacy against *L. amazonensis* strains is scarce. The finding that the strain isolated from the dog is less susceptible to MIL may have implications for clinical veterinary practice. Further studies are needed to ascertain etiological agents causing CVL other than *L. infantum* and their susceptibilities to antileishmanial agents.

## 5 CONCLUSIONS

*L. amazonensis* strains isolated from different clinical forms/hosts displayed functional and biochemical polymorphisms in their LPGs. Qualitative differences with respect to side chain substitutions enabled the description of three types of LPG (I–III). Although three strains (GV02, BA276, and LV78) bearing side chains in their LPGs had higher pro‐inflammatory profiles, a clear correlation was not fully established in murine macrophages. Finally, the lower in vitro susceptibility *L. amazonensis* to MIL warrants further investigation into viscerotropic species affecting dogs and, consequently, the clinical management and therapeutic approaches against CVL.

## AUTHOR CONTRIBUTIONS

**Felipe D. Rêgo**: Formal analysis; investigation; methodology; writing—original draft; writing—review and editing. **Camila d. A. Cardoso**: Methodology. **Paulo O. L. Moreira**: Investigation; methodology; validation. **Paula M. Nogueira**: Investigation; methodology; supervision. **Márcio S. Araújo**: Data curation; formal analysis; methodology. **Valéria d. M. Borges**: Conceptualization; supervision. **Márcia D. Laurenti**: Conceptualization; investigation; writing—original draft. **Daniella C. Bartholomeu**: Investigation; validation. **Alexandre B. Reis**: Investigation; validation. **Rubens L. d. Monte‐Neto**: Formal analysis; methodology; validation; writing—original draft. **Rodrigo P. Soares**: Conceptualization; formal analysis; funding acquisition; investigation; project administration; supervision; validation; writing—original draft; writing—review  and editing.

## ETHICS STATEMENT

Animals were kept in the Animal Facility of the Instituto René Rachou/FIOCRUZ. All procedures were conducted according to the guidelines of the Brazilian College for Experiments with Animals (COBEA‐Law 11.794/2008) and in accordance with animal practice as defined by Internal Ethics Committee in Animal Experimentation (CEUA) (Protocol LW‐32/16).

## Data Availability

The data that support the findings of this study are available from the corresponding author upon reasonable request.
